# Supplementary material for: Significant gap between Point participation and long‑term treatment adherence: An evaluation of ivermectin MDA in the Kwanware‑Ottou persistent onchocerciasis transmission focus, Wenchi, Ghana
Source: PLoS Negl Trop Dis. 2026 Feb 27;20(2):e0013171. doi: 10.1371/journal.pntd.0013171 (PMC12965697; doi:10.1371/journal.pntd.0013171)
Supplement: Attachment — Submitted filename: Response_to_Reviewers.docx [file pntd.0013171.s003.docx]

**Response to Reviewers**
Manuscript ID: PNTD-D-25-00783

Title: Evaluating participation in Ivermectin Mass Drug Administration in the Kwanware-Ottou Persistent Onchocerciasis Transmission Focus in Wenchi, Ghana

Dear Dr. Lopes-Torres and Reviewers,

Thank you for your thoughtful and constructive comments on our manuscript. We appreciate the opportunity to revise and improve our work. Below, we provide a point-by-point response to each comment raised by the reviewers and the editor. All changes have been incorporated into the revised manuscript, which includes a tracked version and a clean copy.

Sincerely,
Rogers Nditanchou, Ph.D.
On behalf of all co-authors

**Reviewers' Comments:**

**Reviewer #1:** The objectives and hypothesis are clear for this study. The data used is partially historical: microfilarial prevalence levels from 1989 and mf and OV16 levels from 2021, and partially self reported by participants surveyed for effective participation. The authors acknowledge that this could potentially result in over reporting in some instances (social desireability), and underreporting in others due to recall bias, which means they could cancel each other out. The statistical methods are appropriate for the data, thogh some sites are poorly sampled, so it might be beneficial to redo the regression model with only well sampled sites to compare odds ratios. I have no ethical or regulatory concerns.

Thank you for your feedback. To address uneven site sampling, we grouped sites into “main” and “satellite,” each with over 100 observations. Predictor categories also met acceptable sample size ranges (8–25), supporting model reliability; see lines 268-269

We ran a sensitivity analysis by comparing models with and without merging a small category (Mining [unauthorised], n = 19) into Cattle Rearing. Results were consistent, justifying its retention as a distinct category due to theoretical relevance.

See lines 269-271; Table 2, line 316-318; and Table 4 lines 351-353

**Reviewer #2:** (No Response)

Reviewer #3: Lines 115-124: Is there baseline information on onchocerciasis endemicity or the pre-control prevalence (%) in Wenchi HD? While I noticed that some data are later presented in Table 2, it may be helpful to briefly mention the baseline status here for context.

Thank you. We have added this in lines 147-164 including Table 1.

Additionally, could the authors clarify how the COVID-19 pandemic affected programme implementation? Was uninterrupted biannual CDTI maintained since 2018, or were there any disruptions?

Thank you for this critical observation. We have now addressed it in the revised manuscript, lines 181–183.

If there was a temporary halt, I recommend discussing its potential implications for onchocerciasis control and elimination progress in both this focus and in Ghana overall in the Discussion section (e.g., see paper "What does the COVID-19 pandemic mean for the next decade of onchocerciasis control and elimination?").

Thank you for this critical observation. We have now addressed it in the revised manuscript, lines 420-431.

Line 123: I appreciate that confidence intervals (CIs) are provided for microfilarial prevalence. I encourage the authors to also report CIs for other key measures in this paragraph, including blackfly infectivity rates if possible. If CIs are not feasible, please consider indicating the sample sizes (e.g., how many individuals or flies were surveyed), as this would help readers assess representativeness.

Thank you for this observation. We have now addressed it in the revised manuscript, lines 149–160; 189-191.

Line 124: Please replace “5.9‰” with “5.9%” and remove the unnecessary period at the end of the sentence.

We have retained the use of “‰” as it accurately reflects the rate per thousand. The additional period has been removed. Lines 184-185.

I also recommend defining abbreviations such as “km” and “CI” upon their first use.

We also appreciate. We have addressed these in lines 159, 161(CI) and lines 166 (km).

Lines 125-127: Given that the transmission focus spans three HDs, were there any periods when different control interventions were implemented in these HDs, or did all three consistently receive annual/biannual CDTI simultaneously? (and vector control?).

Thank you for these critical comments. We have included the intervention history. See lines 170-183.

Figure 1: I suggest including, perhaps in the figure subtitle, the years during which serological and parasitological surveys were conducted, as well as the years of fly collection and larval inspection.

Thank you. We have included the dates of the surveys in legend of Fig. 1.

In the Methods section, it may be informative to cite the range of monthly biting rates or MTP from reference 20, as these data help contextualise the transmission potential. The reported MBRs from reference 20 (5,000–6,000 in Ottu and Kwanware) seem high relative to the moderate baseline endemicity in Table 2. Were the MBRs always this high, or have they fluctuated? Some clarification on the relationship between these metrics would be helpful.

Thank you for this critical contribution. We updated. See lines 184-191.

Indeed, given the persistent transmission and high MBRs, in contrast to the moderate baseline microfilarial prevalence of this focus (e.g., hypo- to mesoendemic), would it be more relevant to explore which community groups may be exposed to the high MBRs (subgroup of the population may be considered hyperendemic at baseline).

Thank you for this critical contribution. We have determined this in our preference study (reference 9). We have updated in lines 158-163; Fig. 1.

Lines 140-142: How was the term “transmission focus” defined? Did the authors follow a specific criterion (e.g., WHO’s 10 km guideline) for this designation?

Thank you for the helpful comment. The Kwanware-Ottou transmission focus was delineated based on a 2021 investigation identifying persistent transmission within a 10 km radius around Ottou. This observation aligns with other studies and WHO guidance which consider a 10–12 km radius as the smallest epidemiological unit for onchocerciasis transmission. We have clarified this in the manuscript and cited the relevant sources. Lines 158-163.

Lines 145-147: The use of GPS satellite imagery to identify settlements and its integration with field and government data is somewhat innovative, commendable methodology.

Thank you. We appreciate this recognition.

Lines 177-178: While I understand the rationale for capping reported MDA participation at 10 rounds, especially since biannual treatment began only in 2018, I suggest clarifying that this may not perfectly reflect years of treatment. The current discussion appropriately notes that 10 rounds may not always correspond to 10 years.

Thank you for the observation. We agree and have clarified in the manuscript. Lines 241-243.

Lines 179-182: The classification of “non-effective” participation as fewer than 10 rounds may be affected by recall and desirability biases. I recommend considering exploratory analyses using shorter thresholds, ideally based on prior literature and your own data collection to further strengthen this aspect.

Thank you for this critical observation. We have addressed this concern in lines 244, 254-256; 326-328 and incorporated relevant exploratory analyses in Table 3 (lines, 330) and Figure 3.

Line 206: Should “Ov16” read “anti-Ov16”? Were participants tested for antibodies to the Ov16 antigen (anti-Ov16), or for the antigen itself (Ov16)? Please clarify and use consistent terminology throughout the manuscript.

Thank you. We have corrected this in the now line 288 and throughout.

Line 207: When referring to “communities,” is this synonymous with the 19 “settlements” described in the Results? Please ensure consistent terminology to aid reader understanding.
Thank you. We have corrected this in the now line 200-202; 294 and throughout

Length of stay in the community: For participants with up to 10 years of residence, were responses capped or excluded if they had not lived in the area for the full duration of MDA implementation? Alternatively, was an exploratory analysis conducted to assess whether these individuals influenced the results? Clarification here would improve the robustness of the findings.

Thank you for the comment. We did not exclude participants based on residence duration. Individuals’ duration in the community contribute to transmission, though the extent depends on length of stay, lines 253–256 and 286-290.

Consecutive ivermectin treatment also affects transmission dynamics. While MDA exposure was not specifically assessed, we examined the impact of visitors, travel, and residence duration on MDA participation. We recognize the potential influence of assessing MDA exposure which need to be considered in future work, lines Table 5, lines 360; 510–517.

**Results**

-Does the analysis presented match the analysis plan?

-Are the results clearly and completely presented?

-Are the figures (Tables, Images) of sufficient quality for clarity?

Reviewer #1: Yes.
The figures are sufficient for the analysis, and clear.

Thank you.

Reviewer #2: (No Response)

Reviewer #3: Line 226: Was age assessed for normality before reporting the mean and standard deviation (SD)? Given the inclusion criteria starts at age 15, the age distribution may be skewed, so median and interquartile range (IQR) might be more appropriate. Please define “SD” as “standard deviation” upon first use.

Thank you. We have now modified the manuscript to median given the age shift from 15 years and more and IQR. See line 301.

Line 213: Table 1 does not require brackets. Additionally, please check for typographical errors such as “participati on” and “co verage,” which appear in my version of the manuscript.

Thank you. We have now updated the manuscript in now table 2. See lines 303.

Table 1: Table 1 addresses two important concepts: effective participation (at least 10 rounds of ivermectin) and point participation (participation in the last round). Many programmes emphasise point participation, but effective participation is often more critical for infection clearance. While recall bias limits this measure’s precision, the distinction is valuable; point participation is relatively high (80.3%) among the eligible population, but effective participation is notably lower (53.4%).

We appreciate your recognition of the distinction between point and effective participation. Now in Table 2 lines 316-318.

Table 2: It is notable that this hypo- to mesoendemic focus at baseline continues to exhibit substantial onchocerciasis transmission (as shown by microfilariae and anti-Ov16 prevalence, 0–30% and 1–19%, respectively); but again, perhaps less suprising given the very high MBRs. Are there particular communities within the area, or subgroups (e.g., those closer to blackfly breeding sites with >400 larvae found, per Figure 1), that have disproportionally higher transmission or disease burden? If so, could this inform a more targeted approach to MDA or surveillance?

Thank you. Yes, we identified this in our previous study and discussed it in the background section. Lines 158-163; Figure 1. This study was nested within this holistic approach. lines 200-201.

We have additional recognised the value of mapping high infection/transmission area for surveillance. See lines 437- 441; 524-526.

If data in Table 2 were sourced from Reference 20, please include a citation in the table.
Thank you. We have now included (and corrected) the reference, now 11. See now Table 1. Lines 155 and Table 3 lines 330

Additionally, what does the hyphen “–” signify in Table 2? Does it indicate missing data? For the Branam community at baseline, the cell is blank, could this be clarified?

Corrected - see now Table 1 (new), lines 159-162

Further, the methodology for assessing microfilariae and anti-Ov16 prevalence is not described in detail. Was microfilarial prevalence assessed in adults? How were skin snips performed (e.g., two samples per person, inverted or classic microscopy)? Which assay was used for anti-Ov16? If these results are to remain in the Results, I suggest adding this methodological detail in the Methods section.

Thank you for these observations. We have updated the now Table 3 and provided clarification, lines 330-337.

Lastly, why is the Ottu community not mentioned in Table 2, given the focus on the Kwanware-Ottu persistent transmission focus? Reference 20 appears to have data for both microfilarial prevalence and anti-Ov16 seroprevalence for Ottu.

Thank you for these observations. We have indicated and justify that effective coverage in Ottou is an outlier and so left out of the correlation analysis. We have provided clarification lines 307-313 and updated the now Table 3, lines 330-337

Line 241: Is there a specific reason for enumerating this paragraph as “1.” without subsequent numbered items? If not, the enumeration can be removed for clarity.

Thank you. We have removed this. Line 338.
Table 3: Were variables assessed for multicollinearity, particularly between ethnic groups and sub-districts, since certain sub-districts may predominantly comprise particular ethnic groups or occupations (e.g., miners in Boase)? The Discussion (lines 308–325) notes that sub-districts may overlap substantially with occupational groups.

Thank you for this critical observation. We have explored this possibility and found no cause for concern. Please refer to lines 266–268 for details.

Lines 247–256: Please provide complete comparisons in the text. For instance, “miners and students or other occupations have significantly lower odds of being in the higher categories” should specify “compared to farmers.” Please clarify the reference categories in sentences about ethnic groups and sub-districts as well.

Thank you. We have updated this. See lines 339-349.

Line 262: Instead of a standalone sentence, please include “(see Table 4)” in brackets.

Thank you. We have updated this. See line 360-361 (now Table 5).

Lines 260–264: The phrase “associations in the same direction” would benefit from clarification. This likely means that the direction of associations for effective and point participation is consistent.

Thank you. We have updated this. See line 355-357.

Additionally, how does this compare to the previous model, given that the explanatory variables are the same?

Thank you. We have updated this. See lines 358-360; Table 5, lines 361-362; 433-441.

Lines 261–262: Is there a reason for the observed differences between effective and point participation models, particularly regarding the “travel category”? Additional explanation would be valuable.

Thank you. We have updated this in the discussion section. See lines 438-441.

**Conclusions**

-Are the conclusions supported by the data presented?

-Are the limitations of analysis clearly described?

-Do the authors discuss how these data can be helpful to advance our understanding of the topic under study?

-Is public health relevance addressed?

Reviewer #1: This manuscript is of public health interest, though additional details on how to further understand this data could be presented. Descriptive methods could be used to explain how the anomalous Ottou site, with very high rates of effective and point participation differs from the other sites.

Ottou reported an effective participation rate of 90.2%, determined to be an outlier. Despite its proximity to Kwanware and similarities in contextual factors, as of now we have no clear explanation to account for this anomaly. We suspect data collection or reporting errors, possibly due to confusion between point and effective participation. Excluding Ottou lowers the overall rate to 52.7%, aligning more closely with other settlements notably Kwanware. See lines 308-314.

Reviewer #2: (No Response)

Reviewer #3: Lines 273–274: The statement, “The exhaustive sampling provided accurate and precise estimates, with negligible sampling error (24),” implies formal testing for accuracy and precision. If such testing was performed, please add the relevant details to the Methods and Results. If not, I suggest revising to a more tentative statement, such as, “…aimed to provide accurate and precise estimates of coverage”

Thank you. We have updated this. See lines 367-368.

Line 279: Rather than “some,” please specify the exact number of settlements (e.g., “ten”).

Thank you. We have updated this. See line 376-378.

Lines 283–285: The phrase “would have been missed during usual CES” suggests certainty. If this is hypothetical, please rephrase to “could have been missed…” or provide supporting evidence.

Thank you. We have updated this. See line 381-382.

Lines 303–304: Clarify how “indicates a more stable model when changing from the treatment category 5–9 times to ≥10 times” relates to the earlier statement in lines 240–256 (“The model is most stable for change from 2 (5–10 times) to 3 (>10 times) categories”). The intervals are defined differently; please ensure consistency or explain the distinction.

Thank you. We have corrected this. See lines 349; 397.

Lines 307–308: Could you clarify why “<10 times” and “≥10 times” is a viable cut-off for effective participation? While this threshold aligns with programmatic guidance, the model explains only 14.5% of the variability, which may represent a normal range for community settings but still relatively low, especially when compared to the point model’s 34.2%.
The threshold of <10 versus ≥10 times for effective participation is supported both programmatically and statistically. Programmatically, it aligns with global guidance that sustained participation—defined as receiving treatment at least 10 times—is necessary for individual and community-level protection. **Statistically, the effective participation model using this threshold explains 34.2% of the variation in outcomes, which is substantially higher than the 14.5% explained by the point participation model and the 8% reported by Breiger et al.** This suggests that the binary categorization not only reflects operational targets but also offers stronger predictive performance, particularly in relation to travel-related adherence patterns. It’s worth noting that the reviewer may have interpreted the model comparison in reverse order.

See results section lines 358-360 and discussion section lines 395-400.

Lines 328–329: Do you have specific recommendations for improving coverage among highly mobile communities (e.g., miners, farmers)? Would strategies such as delivering ivermectin during mining activities, or incorporating satellite data for planning, help improve treatment coverage?

Thank you for the suggestion, which emphasizes the relevance of the study. We have addressed this in the revised manuscript—please see lines 452–464.

Lines 317–342: I commend the thorough limitations section, particularly the discussion of recall and social desirability biases. However, in lines 325–342, caution is needed: while some individuals may have received more than 10 treatments over three decades, interruptions could allow adult worms to resume microfilariae production and sustain transmission. The impact of missing one or two rounds, whether consecutively or intermittently over 10 years, may differ and could be explored further.

Thank you for this critical view. We have now included. See lines 418-432; 511-518

Vector Dynamics and Control Strategies: Given the high biting rates observed (up to 6,000 MBR, per Reference 20), I encourage the authors to discuss their implications. In Togo, such rates were associated with holoendemicity (up to 90% microfilariae prevalence at baseline), yet elimination was nearly achieved through a combination of biannual ivermectin and vector control (see “Reaching Elimination of Onchocerciasis Transmission with Long-term Vector Control…” and “Impact of ivermectin and vector control on onchocerciasis transmission in Togo…”). Reference 20 notes that Ghana also implemented vector control during OCP, including in rivers near Kwanware and Ottou, but vector control is not mentioned in the manuscript. Would the authors recommend re-implementing focal vector control (e.g., ground larviciding or Slash and Clear), alongside improved CDTI, for persistent foci? Might further surveys soon after multiple years of biannual CDTI provide greater insight into trends?

Thank you for highlighting this important point. We agree that the high biting rates observed in Kwanware and Ottou suggest intense transmission potential. We have now expanded the discussion to reflect the implications of these rates, referencing successful elimination efforts in Togo and the historical vector control in Ghana. We also acknowledge the potential value of reintroducing focal vector control (e.g., larviciding or Slash and Clear) alongside improved CDTI, and the importance of follow-up surveys after sustained biannual treatment. Please see revised Discussion section, lines 465–478.

Policy Recommendations: A brief policy recommendation paragraph before the conclusion would be helpful. Besides using elimination modelling, do the authors suggest introducing prospective surveillance for effective participation to reduce recall bias, or other surveillance improvements? As well as some of the recommendations I suggsted earlier.

Thank you for this helpful suggestion. We agree that a brief policy recommendation section strengthens the manuscript. We have now added a paragraph before the conclusion summarizing key recommendations, including the need for prospective surveillance of effective participation to reduce recall bias, consideration of focal vector control in persistent foci, and the use of elimination modelling to guide localized strategies. Please see lines 528-543

Mobility and Reinfection Risk: In lines 215–216, it is noted that “many unoccupied temporal shelters” are used by farmers, miners and cattle rearers. Do these groups reside within or outside the transmission focus? If outside, could they act as a source of reinfection to areas in Ghana where elimination is nearly achieved?

Thank for these insightful thoughts. They are inside but similar places and foci could exist within the larger Tano-Aubra OTZ that can cause reintrotuction unless the strategy is implemented wider. See lines 465-464.

Contextualisation: How does the Kwanware-Ottou focus fit within the broader context of onchocerciasis control and elimination efforts in Ghana?

Thank you. we have mentioned that similar areas may exist and/or because of movement it can cause persistent transmission in Kwanware-Ottou and other foci or reintroduce infection in areas nearing elimination unless a broader approach is adopted. See lines 458-464; 519-527.

**Editorial and Data Presentation Modifications?**

Use this section for editorial suggestions as well as relatively minor modifications of existing data that would enhance clarity. If the only modifications needed are minor and/or editorial, you may wish to recommend “Minor Revision” or “Accept”.

Reviewer #1: It appears that Dagaaba is misspelled as Dagaarba throughout the manuscript. Please ensure that this spelling is appropriate and not a lesser used or historical name for this group.

Thank you. We have corrected this in line 345 and throughout.

Reviewer #2: (No Response)

Reviewer #3: Background Section

Line 68: Consider whether the abbreviation “Ov” is necessary. In lines 61–77, you use the standard abbreviation “O. volvulus,” which is more common in the literature.

Thank you. We have corrected this in line 75 and throughout.

Lines 69–70: It would strengthen your argument to add a reference for the onchocerciasis burden.

Thank you. We have corrected this in line in lines 75-83.

Additionally, consider mentioning that the burden of onchocerciasis has recently been associated with neurological complications, such as onchocerciasis-associated epilepsy.

Thank you. We have included this in lines 76-80.

Lines 75–76: Please clarify whether “therapeutic coverage” refers to the overall population or to the eligible population (likely the latter, given the scope of the manuscript).

Thank you. We have included this in lines 108-109.

Lines 74–77: The explanation that ivermectin must be administered for several years to match the reproductive lifespan of the adult worm is helpful. For clarity, please add that ivermectin is microfilaricidal, it kills the larvae (microfilariae) of O. volvulus, not the adult worms, therefore the long treatment.

We have now corrected this. Line 101-103.

Also, the 85% coverage threshold for eligible population is typically cited for the onchocerciasis elimination programme in the Americas, while 80% is the standard for Africa (see Guidelines for stopping mass drug administration and verifying elimination of human onchocerciasis: criteria and procedures). Is there a reason for using 85% as the benchmark for Ghana in this article? If so, please clarify. That said; I agree that higher coverage is always preferable, especially in such a high-transmission focus.

Thank you. We have now corrected this to 80%. Line 103 and throughout.

Lines 81–82: When stating, “To assess this, it is recommended that coverage evaluation surveys (CES) be conducted every 1–3 years at the evaluation unit level,” please specify who recommends this and include a reference.

We have now corrected this. Line 110-112

Lines 84–85: These lines, while clearer, appear to partially repeat information from lines 80–81. Consider consolidating for clarity and conciseness.

We agree. This is now removed. Line 116.

Lines 87–88: Although APOC will be familiar to expert readers, it would be helpful in earlier paragraphs, when introducing onchocerciasis control, EOT, Ghana, etc., to mention that onchocerciasis control in Africa began with OCP and later APOC.

Thank you. We have added this in lines 107-110.

Line 89: Regarding the statement, “little consideration has been given to this kind of evaluation thereafter (13),” please clarify what “thereafter” refers to. Does it mean after a programme in the APOC area reaches 8 years of MDA, or after a specific year when such surveys were mainly conducted?

Thank you. We have clarified this in lines 115-119.

Lines 91–93: Since geographic and therapeutic coverage are key components of the manuscript, I recommend briefly defining how each is determined in the introduction.

We have added this. Line 98-104.

**Summary and General Comments**

Use this section to provide overall comments, discuss strengths/weaknesses of the study, novelty, significance, general execution and scholarship. You may also include additional comments for the author, including concerns about dual publication, research ethics, or publication ethics. If requesting major revision, please articulate the new experiments that are needed.

Reviewer #1: The manuscript “Evaluating participation in Ivermectin Mass Drug Administration in the Kwanware-Ottou Persistent Onchocerciasis Transmission Focus in Wenchi, Ghana” discusses current shortfalls in ivermectin MDA efforts in treating Onchocerciasis, a neglected but very treatable filarial parasite in the Wenchi health district. Both historical data (microfilarial prevalence levels from 1989 and mf and OV16 levels from 2021) are compared against survey data on usage of more than 10 administrations of ivermectin (effective), compared against ivermectin administration in the most recent round of CDTI.

Thank you for your positive and constructive feedback on our manuscript.
Overall the manuscript is well written and the statistical methods are appropriate for the types of data used in the study. The results should be of interest to those in public health and those in the filarial community
We appreciate your recognition of the clarity, relevance, and methodological rigor of the study.

I have only minor comments:

- Curiously, some regions exceed the recommended levels of effective participation in MDA (Ottou, Kwadom Joboy).

Thank you for this observation. We have discussed this in lines 307- 313; and 495-498.

In the case of Ottou it is excluded from the analysis as an outlier. It nevertheless stands as an anomaly. Based on the regression model it would still be interesting to see how Ottou fits the model.

Thank you for this observation. We have detailed our reasoning in lines 313-318 and 335-342.
- It appears that Dagaaba is misspelled as Dagaarba throughout the manuscript. Please ensure that this spelling is appropriate and not a lesser used or historical name for this group.

Thank you. We have corrected this in line 344 and throughout.

Reviewer #2: The authors evaluated participation in ivermectin mass drug administration (MDA) for onchocerciasis in the Wenchi district of Ghana. The study highlights that low participation rates and limited geographical (settlement-level) coverage contribute to persistent transmission of the disease.

Thank you for seeing value to this. We demonstrated that low participation and limited settlement-level coverage are key drivers of persistent transmission. Our findings support the need for more targeted and geographically inclusive MDA strategies. We have discussed this in the discussion section. Lines 518-535.

1. The manuscript focuses solely on participation, but does not provide any information on compliance, which is essential to fully assess the effectiveness of CDTI efforts.

We have defined participation as actually swallowing ivermectin during distribution, which aligns with frequent concept of compliance. However, by framing it as participation, we shift the responsibility from solely the beneficiary to all stakeholders, including program implementers and community drug distributors. See lines 228-238.

2. Annex 1 is referenced but not included in the submission.

Thank you we have now included Annex 1 (Appendix 1). Line 768.

3. The manuscript lacks a broader discussion comparing findings with those from other countries, and especially from other districts or regions within Ghana. Several studies have been published on participation and compliance with CDTI, and integrating these comparisons would help identify key lessons to improve program success and support onchocerciasis elimination.

We appreciate the suggestion. Our study defines participation as swallowing ivermectin during distribution, which aligns with compliance. Therefore, our focus was not on the participation–compliance gap but on effective and point treatment uptake (participation) within a delineated transmission focus. See lines 228-238

4. Figure 1: An overview map of Ghana should be included before the study area map to help readers understand the location of the Wenchi district within the national context.

Thank you. we have included this in Figure 1.

5. Table 1: The asterisk (*) next to "Ottou" needs to be explained in a footnote or legend for clarity.

Thank you we have included the explanation in the legend of now Table 2. See lines 316-318.

6. In the Results section, it would be helpful to briefly summarize the key findings from the tables in one to two sentences. This would allow readers to quickly grasp the main take-home messages.

Thank you we have included this throughout the result section, lines 291-361.

Reviewer #3: This is an interesting and timely paper, particularly relevant as sub-Saharan Africa advances toward the ambitious goal of onchocerciasis transmission elimination and eventual eradication. I appreciate that the authors provided a version of the manuscript with numbered lines, which facilitated referencing specific points throughout this review.
One of the main strengths of the paper lies in its thoughtful discussion of the limitations inherent in reporting geographic and therapeutic coverage at the district or larger administrative levels, an issue that often results in missing small settlements that may act as persistent pockets of transmission. The authors also draw attention to the significant impact of intermittent ivermectin intake: missing several years of treatment may prevent infection clearance, a crucial and sometimes underappreciated challenge in longstanding onchocerciasis control efforts across sub-Saharan Africa. These points are known but relevant, especially in the context of a high-endemicity area in Ghana where nearly three decades of ivermectin distribution have not interrupted transmission.

Overall, the manuscript is comprehensive and demonstrates critical thinking, offering a wealth of information on the transmission focus under study. The inclusion of detailed epidemiological and programmatic data, along with reflections on the challenges and implications for control, is commendable and adds value to the field. Such thoroughness is not always present in programmatic literature and will be appreciated by both researchers and policy-makers, especially after answering some of review queries for complementary information.

The study’s key strengths include its exhaustive approach to data collection, the integration of both epidemiological and entomological findings, and the critical reflection on programmatic and methodological limitations. The discussion is balanced and the policy recommendations are well founded, although they could be further expanded, especially regarding strategies to address persistent transmission in hard-to-reach communities and within Ghana.

We appreciate the recognition of the manuscript’s strengths and relevance to ongoing elimination efforts. We have expanded the policy recommendations to include targeted strategies for addressing persistent transmission in hard-to-reach communities in Ghana, as suggested. See lines 518-542.

Some minor points, such as the need for more consistent terminology, clarification of methods, and a more cautious interpretation of some outcomes, are addressed in detail in the specific comments. While part of the data has been publsihed before, there are no concerns about research ethics or dual publication. Overall, I believe the manuscript makes a significant contribution to the understanding of onchocerciasis control and surveillance in highly endemic, resource-constrained settings of West Africa.

I recommend publication after revision, with attention to the specific methodological clarifications and policy recommendations outlined in my comments.

Thank you for the recommendation. We have addressed the minor edits and clarified the methods as indicated in the relevant sections above. We appreciate your support and the valuable contribution to strengthening the manuscript.

PLOS authors have the option to publish the peer review history of their article ([what does this mean?](https://url.uk.m.mimecastprotect.com/s/1C37CzmnAf8XnoNTWcDi9HzHJ?domain=track.editorialmanager.com)). If published, this will include your full peer review and any attached files.

If you choose “no”, your identity will remain anonymous but your review may still be made public.

**Do you want your identity to be public for this peer review?** For information about this choice, including consent withdrawal, please see our [Privacy Policy](https://url.uk.m.mimecastprotect.com/s/jJf4CAnmrtrxVAJt1fviGpWJ3?domain=track.editorialmanager.com).

Reviewer #1: No

Reviewer #2: No

Reviewer #3: **Yes:** Luís-Jorge Amaral

[NOTE: If reviewer comments were submitted as an attachment file, they will be attached to this email and accessible via the submission site. Please log into your account, locate the manuscript record, and check for the action link "View Attachments". If this link does not appear, there are no attachment files.]

**Figure resubmission:**

While revising your submission, please upload your figure files to the Preflight Analysis and Conversion Engine (PACE) digital diagnostic tool, [https://pacev2.apexcovantage.com/](https://url.uk.m.mimecastprotect.com/s/cfPRCBgnvsPxA5JCXhBi2TumB?domain=pacev2.apexcovantage.com). PACE helps ensure that figures meet PLOS requirements. To use PACE, you must first register as a user. Registration is free. Then, login and navigate to the UPLOAD tab, where you will find detailed instructions on how to use the tool. If you encounter any issues or have any questions when using PACE, please email PLOS at [figures@plos.org](mailto:figures@plos.org). Please note that Supporting Information files do not need this step. If there are other versions of figure files still present in your submission file inventory at resubmission, please replace them with the PACE-processed versions.

**Reproducibility:**

To enhance the reproducibility of your results, we recommend that authors of applicable studies deposit laboratory protocols in protocols.io, where a protocol can be assigned its own identifier (DOI) such that it can be cited independently in the future. Additionally, PLOS ONE offers an option to publish peer-reviewed clinical study protocols. Read more information on sharing protocols at [https://plos.org/protocols?utm_medium=editorial-email&utm_source=authorletters&utm_campaign=protocols](https://url.uk.m.mimecastprotect.com/s/wme7CDR0xij7MlrIXiPijLByr?domain=plos.org)

*In compliance with data protection regulations, you may request that we remove your personal registration details at any time.* [*(Remove my information/details)*](https://url.uk.m.mimecastprotect.com/s/bCGKCElPyf1wn5os0s2i7iZLi?domain=track.editorialmanager.com)*. Please contact the publication office if you have any questions.*
