# Supplementary material for: Significant gap between Point participation and long‑term treatment adherence: An evaluation of ivermectin MDA in the Kwanware‑Ottou persistent onchocerciasis transmission focus, Wenchi, Ghana
Source: PLoS Negl Trop Dis. 2026 Feb 27;20(2):e0013171. doi: 10.1371/journal.pntd.0013171 (PMC12965697; doi:10.1371/journal.pntd.0013171)
Supplement: Attachment — Submitted filename: response_letter_v2.docx [file pntd.0013171.s004.docx]

30th January 2025

**Response to Reviewers**
PNTD-D-25-00783R1

Title: Evaluating participation in Ivermectin Mass Drug Administration in the Kwanware-Ottou Persistent Onchocerciasis Transmission Focus, Wenchi, Ghana

Dear Dr. Lopes-Torres and Reviewers,

Thank you for your thoughtful and constructive comments on our manuscript. We appreciate the opportunity to revise and improve our work. Below, we provide a point-by-point response to each comment raised by the reviewers and the editor. All changes have been incorporated into the revised manuscript, which includes a tracked version and a clean copy.

Sincerely,
Rogers Nditanchou,
On behalf of all co-authors

**Methods**

Reviewer #2: (No Response)

Reviewer #3: Methods:
1) Line 154: “Microfilariae” does not need a hyphen in the manuscript text.

Thank you. This now removed. Line 151.

2) Table 1 and related text: It would be helpful to clarify when vector control started in this health district. Was it in nineteen seventy four, as in Ghana overall If so, the prevalence reported for nineteen eighty would not correspond to a true baseline, which suggests hyperendemicity and high transmission potential before control. This clarification is mentioned later around paragraph 170, but it would be very useful to state it earlier in the table legend or in the first methods paragraph with the epidemiological indicators.

Thank you. We have added in the legend that “Vector control in the Tain River began in 1976 under the OCP, so the prevalence measured in 1980 may not represent the true baseline level. This suggests hyperendemicity and high transmission potential at baseline ”. Lines 154-156

3) First methods paragraph and Table 1: Just an observation, the increasing trend in prevalence in Kwanware and Ottou, although based on recent small sample sizes, suggests high transmission potential, compounded by sub-optimal effective CDTI as you discuss in the manuscript. It could be interesting to conduct a brief human landing catch survey in this location.

Thank you for your observation. We agree that the increasing prevalence in Kwanware and Ottou, despite the small sample sizes, may indicate high transmission potential compounded by sub-optimal CDTI. Regarding your suggestion, we have already elaborated the results of a human landing catch survey in our previous study, which is referenced in lines 184–192.

Figure 1 is very clear and visually informative.

Thank you for the complement.

4) Line 184: It is not certain that all readers will immediately recognise the meaning of “%0” notation. I would consider briefly defining it the first time it appears.

Thank you for pointing this out. The “‰” notation indeed stands for per thousand. We have now clarified this at its first appearance in line 184 to ensure all readers understand the meaning.

**Results**

Reviewer #2: (No Response)

Reviewer #3: Results:
Figure 2 is also clearly presented and easy to interpret.

Thank you for the complement.

1) Table 2: For Ottou, please consider reporting the exact p-value if possible. At present it is written as “less than zero point two four three” or similar. The "<" may just be a typo.
Thank you for highlighting this. You are correct — the “<” notation was a typographical error. We have corrected this in Table 2 by reporting the exact p-value for Ottou.

2) Lines 305 and 306: You state that the young population had a coverage of less than fifty five percent. If available, please provide the precise percentage.

Thank you for pointing this out. We have updated the manuscript in lines 312–313 to include the precise percentage: *the younger age group (15–24 years) had an effective coverage of 50.8%*.

3) Line 327: Consider whether “microfilaria prevalence” should be “microfilarial prevalence” for consistency of terminology.

Thank you for this helpful suggestion. We agree that “microfilarial prevalence” is the more consistent terminology. We have revised the text at lines 331-332 accordingly.

4) Lines 307 and 322 Ottou as an outlier: I am not entirely convinced that Ottou should be excluded from the analyses as an outlier. While the sample size is indeed small and neighbouring settlements have lower prevalence, this needs careful consideration. Either
a) the population in Ottou truly has high point and longitudinal participation in ivermectin due to their location or knowledge of its importance, and prevalence is driven by high biting rates combined with lower coverage in nearby settlements, or
b) the method used may be less reliable when sample sizes are very small for example with only nineteen individuals, due to biases such as recal bias.
It is likely that both mechanisms contribute, although the first may be particularly important. There may also be additional, less obvious reasons. Importantly, onchocerciasis transmission is highly heterogeneous, in contrast to infections such as influenza where exposure is more homogeneous. Some settlements often experience much higher blackfly biting rates. For this reason, I would still consider including Ottou in the analyses, perhaps with an appropriate note of caution.

Thank you for these critical insights. The definition of an outlier is primarily statistical We have now included Ottou in Table 3 and provided a detailed discussion in lines 444–456 as you suggested:

“Ottou settlement shows both high point and effective participations in ivermectin treatment (90.2% and 97.6%, respectively; Table 2), yet infection levels remain high (40.0%; Table 3). Although statistical analysis identified Ottou’s effective coverage as an outlier and it was excluded from the correlation analysis, this finding warrants careful interpretation. Onchocerciasis transmission is highly heterogeneous (51), unlike infections such as influenza where exposure is more uniform (52). The heterogenicity in Ottou and surrounding is likely due to high biting rates, as previously noted (15), combined with the lower coverage in nearby settlements (15). Alternatively, reliability of reported participation might have been affected by the very small sample size—only 41 individuals—along with potential biases such as recall bias or conflation between recent CDTI round and those conducted in the more distant past. However, these explanations are not supported by observations in neighbouring Kwanware, which shares similar population characteristics but has much lower effective participation and similarly high infection levels (Table 3). Therefore, it appears that the most likely explanation is conflation and/or biases—most notably social desirability bias (53).”

5) Table 3 participation: Please check the proportions for cumulative participation. Several entries appear to exceed one hundred percent when summed by row. For instance 21 participants out of 52 is not 65.3%, and 121 out of 353 is not 27.1%.

Thank you for catching this error. We have now thoroughly revised Table 3 to ensure that all percentages are consistent with their numerators and denominators, and that row totals do not exceed 100%.

6) Table 3 microfilarial prevalence and anti-Ov16: For the values given in brackets for microfilarial prevalence and anti-Ov16, please specify explicitly in the Table what these represent for example 95% confidence intervals.

Thank you for pointing this out. We have now specified in the column heading that these represent the 95% Confidence Intervals.

7) Table 4: “Cattle rearing/” can be simplified by removing the slash?

Thank you. We have now corrected this.

8) Given the abundance of rivers in the study area, fishing is mentioned briefly around line 209. If fishing is a relevant occupation in this population, please clarify whether it was considered as a variable and if not (or no one reported the profession?), whether this potential heterogeneity in exposure is adequately captured under related high-risk occupations such as farming.

Thank you for this observation. We have clarified in Table 4 legend that no participants reported fishing as their occupation. Line 355.

**Conclusions**

Reviewer #2: (No Response)

Reviewer #3: Discussion:
1) Lines 375 to 377: I would be cautious about emphasising the coverage estimates from the 19 settlements too strongly, since some settlements are very small and the estimates may be sensitive to a few households being missed or not targeted. This is more of a nuance than a requested change, as the current text is very acceptable, and indeed the authors express they view well of going into detailed data of onchocerciasis exposure and control.

Thank you for this important observation. We have now incorporated this reflection into the discussion, specifically in lines 381-385.

We now state in the text “These coverage estimates warrant cautious interpretation. Because some settlements have very small populations, the exclusion or mistargeting of just a few households can markedly affect the calculated coverage, potentially overstating or understating true participation. This issue is particularly relevant in the context of elimination efforts that aim to ensure no one is left behind”.

2) Please define the acronym CDD the first time it appears on the main text.

Thank you. We have included this in line 234.

3) Suboptimal coverage: One question that remains is whether suboptimal coverage in some settlements is mainly due to a lack of willingness to take ivermectin at that round, to people being absent or working when distributors visited, or to CDDs moving through too quickly and missing households. A short reflection on these potential mechanisms would add useful context. I might have missed it in the discussion?

Thank you for this valuable suggestion. We have now incorporated this perspective into the discussion, specifically in paragraph 457 (lines 477–481).

We now state in the text. “A critical issue is determining whether suboptimal coverage in certain settlements is driven more by limited willingness to take ivermectin in that round or by CDDs not reaching all households, either because they moved too quickly or inadvertently missed some homes. As discussed earlier, both mechanisms operate simultaneously, although their effects differ between settlements. Future assessments of CDTI coverage should explicitly consider these interacting factors.”

4) Lines 412 and 413: A reference could be needed to support the statement in these lines.
Thank you we have included this in line 421.

5) Closing remark: Overall, the manuscript is a clear and valuable account of Ghana and West Africa efforts to eliminate onchocerciasis while addressing persistent transmission. The analysis of effective participation is particularly informative and underlines the importance of heterogeneity in ivermectin uptake, in parallel with heterogeneous exposure to blackfly bites. The attention to mobile populations is also timely and relevant, since these groups may increasingly act as reservoirs and threaten elimination goals.

Thank you for your complement.

**Editorial and Data Presentation Modifications?**

Use this section for editorial suggestions as well as relatively minor modifications of existing data that would enhance clarity. If the only modifications needed are minor and/or editorial, you may wish to recommend “Minor Revision” or “Accept”.

Reviewer #2: (No Response)

Reviewer #3: Abstract:
1) Line 35: Please consider also reporting confidence intervals for the main quantitative results, especially for effective participation coverage.

Thank you. We have corrected this. Lines 35-37.

Author summary
2) Line 47: The expression “onchocerciasis public health challenge” resembles “public health problem”, which accurately describes the situation in West Africa before OCP. Since onchocerciasis-related morbidity, such as blindness, is now mostly suppressed in Ghana at the moment, it might be more precise to say that “transmission persists in parts of Ghana” or similar wording.

Thank you. We have corrected this line 48.

3) Line 48: There is a missing full stop at the end of the sentence. Otherwise, the author summary reads clearly and is easy to follow.

Thank you for the complement. We have included the full stop. Line 49.

Background:
4) Line 83: A reference appears to be missing for the statement in this line.

Thank you. We have now included references, lines 79-80.

5) Lines 85 and 86: While it is correct that OCP initially relied on vector control, ivermectin delivery became very important from the early nineteen nineties, in some areas even replacing vector control, still during OCP. Therefore, as you explain later in the manuscript, OCP also relied on ivermectin in its later phases. The sentence would benefit from reflecting this evolution more explicitly.
Thank you. We have made this adjustment line 83-84.

6) Lines 91 and 92: This sentence could be strengthened by citing the relevant APOC report that documents elimination as a public health problem being achieved.

Thank you. We have now included. Lines 88-89.

7) Lines 106 and 107: A reference is needed for the reproductive lifespan of the parasite. Otherwise, this paragraph now reads well and is informative.

Thank you. We have included this. Line 105-107

**Summary and General Comments**

Use this section to provide overall comments, discuss strengths/weaknesses of the study, novelty, significance, general execution and scholarship. You may also include additional comments for the author, including concerns about dual publication, research ethics, or publication ethics. If requesting major revision, please articulate the new experiments that are needed.

Reviewer #2: The authors have addressed all raised questions and revised the manuscript accordingly. I therefore support publication.

Thank you for the support.

Reviewer #3: The revised manuscript reads as a very informative contribution and a strong testament to the long term efforts in Ghana and West Africa to eliminate onchocerciasis despite persistent transmission in some areas. The methodology used to quantify effective participation is particularly insightful and highlights important heterogeneity in ivermectin uptake, which is meaningful given that exposure to onchocerciasis is also heterogeneous. The discussion of mobile populations is especially relevant, as these groups may increasingly act as reservoirs and jeopardise elimination efforts as transmission becomes more focal.

Thank you for the complement.
